# Does transparency matter? The moderating effect of algorithm transparency on social influence in investment decisions

**DOI:** 10.3389/fpsyg.2026.1777758

**Published:** 2026-04-09

**Authors:** Wei Chang, Jingjing Yu

**Affiliations:** 1 School of Finance, Shanghai Lixin University of Accounting and Finance, Shanghai, China; 2 School of Finance, Capital University of Economics and Business, Beijing, China

**Keywords:** algorithmic transparency, investment decision, perceived autonomy, self-determination theory, social influence

## Abstract

**Introduction:**

With the rapid development of intelligent investment advisory platforms, the interaction between algorithmic transparency and social influence in investment decision-making remains underexplored. Based on Self-Determination Theory, this study investigates how algorithmic transparency moderates the effect of social influence on investment decisions, with perceived autonomy as a proposed mediating mechanism.

**Methods:**

Two progressive online experiments were conducted (total *N* = 400, recruited via the Credamo platform). Study 1 employed a 2 (Social Information: Present/Absent) × 2 (Algorithm Transparency: High/Low) between-subjects design (*n* = 240). Study 2 (*n* = 160) additionally measured perceived autonomy as a mediator. Hypotheses were tested using two-way ANOVA and Hayes’ PROCESS macro (Model 8, 5,000 bootstrap samples).

**Results:**

Social influence significantly increased investment intention (F(1,236) = 24.87, *p* < 0.001, η² = 0.095). Algorithmic transparency negatively moderated this effect, reducing the impact of social information by approximately 34.7% under high transparency conditions (F(1,236) = 4.89, *p* = 0.028). Exploratory analysis suggested the moderating effect was weaker among investors driven by social connection motives. Perceived autonomy partially mediated the moderating effect, with a mediation proportion of 34.8% (indirect effect = −0.031, 95% CI [−0.055, −0.008]).

**Discussion:**

Algorithmic transparency reduces investors’ reliance on social information primarily by enhancing perceived autonomy. The effect is most pronounced among nonprofessional investors with low-to-moderate financial literacy. These findings offer empirical support for transparency-oriented fintech platform design and regulatory policy formulation.

## 1 Introduction

The rapid development of financial technology is profoundly reshaping the way and environment of individual investment decisions. As of 2024, the global assets under management by robo-advisors have reached US$1.8 trillion, and are expected to exceed US$3.5 trillion by 2028 (Zhu et al., 2024). In the Chinese market, internet wealth management platforms such as Ant Fortune and Tiantian Fund have hundreds of millions of registered users, and personal investment selection supported by algorithms has become a normal practice (Li et al., 2022). At the same time, the social aspects of investment platforms have become conspicuous, as shown by the extensive adoption of social components such as mutual portfolios, co-investment capabilities, and investment communities, which have a role in shaping the investment decisions of participants through peer effects (Yoon and Oh, 2022). The resulting complex information context with intertwined algorithmic and social information impacts the investment decision-making behaviors of participants.

In this context, a related issue, algorithmic transparency, is being increasingly studied in academic and regulatory circles. Rules like the EU’s Artificial Intelligence Act or China’s Regulations on the Management of Algorithmic Recommendations for Internet Information Services make algorithmic transparency mandatory. Related research work has found that algorithmic transparency, or how well platforms disclose recommendation logic to users, is a critical determinant of users’ trust and acceptance of AI outcomes (Grimmelikhuijsen, 2023; Smith et al., 2025). Nevertheless, in an investment context, the function of algorithmic transparency may be more complex: if users are exposed to two sources of information at a time—a recommendation algorithm and a friend’s investment choice, then how will transparency influence users in processing and using social information? The answer has important theoretical and practical implications in understanding decision-making processes in digital investment spaces.

The antecedents of investment behavior have been investigated in existing research. For instance, in research related to social influence studies, two mechanisms that influence investment behavior have been identified as social learning and social utility (Bursztyn et al., 2014). Social learning operates through informational transmission wherein investors infer asset quality by observing others’ investment choices—for instance, when knowledgeable friends purchase a particular fund, this signals favorable fundamentals or prospects. Investors learn about asset quality through observing investment decisions made by other investors, which is significant when information asymmetry is experienced (Heimer, 2016). Social utility operates through relational mechanisms wherein investors derive non-financial psychological benefits from coordinated investment behavior—such as shared conversation topics, strengthened group identity, and conformity comfort. Investment behavior serves as a means of satisfying social connection; investors derive a non-monetary gain through owning similar assets as those of their friends because of topics, identity, and feelings of belonging (Kulchania, 2023). Research has included empirical evidence about peer influence having a significant effect on different financial behaviors and mentalities related to the stock market investment (Heimer, 2016; Rashid and Said, 2024). In relation to transparency in algorithms and related research, research has indicated that transparency is significant in building user trust since it improves explainability but that over-transparent algorithms can exhibit information overload or even challenge user confidence in algorithms (Watkins and Woods, 2024; Weber et al., 2024; Zhu et al., 2023; Zhu et al., 2024). Other research related to transparency has investigated effects of transparency on fairness and legitimacy of algorithms perceived by users and has investigated heterogeneous reactions of different groups of users to transparency (Li et al., 2023; Martin and Waldman, 2023; Narayanan et al., 2024; Starke et al., 2022).

Despite the productive outcome of the two streams of research mentioned above, the relationship between algorithmic transparency and social influence has not been examined in the literature yet. This represents the theoretical gap that this study will address. If platforms offer not only algorithm-driven suggestions of investments (with a different level of transparency) but also social information (friends’ behavior in investments), how do people use these two pieces of information? Does a higher level of transparency boost or reduce the power of social influence? Are there national or international differences in the governance of the two social learning mechanisms and of social utility? The response to these questions would require an intersection of the two streams of research: algorithm-driven decision-making and social influence.

This research aims to establish a theoretical basis through the lens of Self-Determination Theory to explore the moderation of algorithmic transparency on social influence and its respective mechanisms. Self-Determination Theory is known to confer importance to three irrevocable psychological needs: autonomy, competence, and relatedness (Deci and Ryan, 2000). This theoretical concept is appropriate to explain the relationship between algorithmic usage and social aspects because algorithmic transparency is capable of influencing users’ reliance on external information through their perception of autonomy; while social learning and social utility are associated with competence (enhanced decision-making capabilities through external information) and relatedness (sustaining social ties through mutual investments), respectively (Baek and Kim, 2023; Fan and Liu, 2022; Puntoni et al., 2021).

The theoretical significance of this research is shown in three ways. The research integrates algorithm transparency into the theoretical framework of social influence and investment decision-making, which can help illustrate the mechanisms under which algorithmic and social forces interact in the digital investment context, and break through traditional boundaries in the research of behavioral finance. In particular, this research highlights the promotions and inhibition of social utility and social learning through transparency, which can help advance our understanding of peer heterogeneity and provide a fresh angle for interpreting variations in social influence outcomes across different settings. Finally, this research transplants a theory from psychology and applied it in a decision-making scenario facilitated by AI technology, which can help clarify a new linkage between transparency and this decision-making scenario and advance applications of this theory in human-computer interaction research. In practice, this research can provide empirical supports for FinTech product designs related to transparency and serve as a benchmark for evaluation related to transparency regulation in FinTech platforms.

## 2 Theoretical background and hypothesis development

### 2.1 Social influence and investment decisions

The importance of social influence in making investment decisions has also been validated in a variety of studies. Theoretically speaking, social influence rests on two models: social learning and social utility (Bursztyn et al., 2014).

The social learning mechanism focuses on the informational function of other people’s behavior. In financial markets, because of information asymmetry and bounded rationality, individual investors cannot obtain and process all financial information independently (Heimer, 2016). In such a situation, learning from other people’s behavior may have a great informational function; for example, if rational investors select a particular asset, this may imply that the selected asset is of great investment value. This mechanism was discovered in financial markets through a clever experimental study; when participants were informed of their friends’ “intent to invest in” a particular asset, there was a sharp increase in the participants’ intention to invest, which partly stemmed from the informational inference rather than the social factor alone (Bursztyn et al., 2014). The impact of social learning mechanism in financial markets became greater in the era of social media, which greatly influenced the dissemination of investor attitudes, especially the formation of “herd behavior” (Chen et al., 2024; Yoon and Oh, 2022).

The social utility mechanism is concerned with the social role of investment activity. Existing studies related to the problem show that investors can potentially obtain non-monetary utility by investing in the same stocks as their friends. The potential sources of social utility include: shared topics of financial investing, which encourage socializing; shared financial investing decisions, which enhance social identities; and consistency with the reference group, which provides social comfort (Fan and Liu, 2022). Experiments have shown that when participants were informed that their friends “actually held” a particular asset, participants’ intentions to invest increased even more than when participants were informed that their friends “planned to invest,” an incremental effect of social relationships which cannot be attributed to the transmission of information, thus highlighting the role of social utility (Bursztyn et al., 2014). Evidence on retirement savings also suggests the role of social utility, where social influence on personal savings is enacted via social interactions, social relationships, and social identity, with stronger effects in more intimately related social groups (Duffee, 2023).

Based on the above theoretical analysis and empirical evidence, this study proposes the following hypothesis:

*Hypothesis 1*: Friends' investment choices (the presentation of social information) positively influence individual investment willingness. It is important to note that a distinction has been made in the previous studies between two forms of social information: “already held” and “intended to invest” information, and that the latter has a greater influence (Bursztyn et al., 2014). It should be noted that this study will focus on the most frequent “already possessed” social information context.

### 2.2 The concept and effects of algorithmic transparency

Algorithmic transparency is defined as “the degree to which a system is able to reveal its underlying decision-making logic, basis, or process to users” (Grimmelikhuijsen, 2023). For the area of robo-advisory, transparency can exist on different levels, namely the presentation level of recommendation outcomes, explanation level for recommendation justification, and explanation level for underlying logic of algorithms (Zhu et al., 2024). From related studies, it was identified how transparency exists on different dimensions, namely outcome transparency (what) and process transparency (how), where it affects different aspects on user trust and adoption (Weber et al., 2024; Zhu et al., 2024).

The positive impact of algorithmic transparency has been verified by different research studies. The users’ level of understanding and predictability concerning algorithmic outputs improves with algorithmic transparency, resulting in enhanced trust (Grimmelikhuijsen, 2023; Starke et al., 2022). In the realm of public decision-making, it was verified that explaining how a particular algorithm disapproved a particular input would substantially boost public confidence and acceptance pertaining to automated decision-making processes (Park and Young Yoon, 2025). The financial services industry views algorithmic transparency as a critical element to attain user trust and enhance the acceptance level of robo-advisory services (Lin et al., 2024). The results from the meta-analysis study denote a prominent positive correlation between user adoption intention and algorithmic explainability (Weber et al., 2024).

Nevertheless, the implications of transparency do not follow a linear progression. It has been supported that complex explanations may cause overload of information, hence negatively affecting the experience of the users (Roh et al., 2023). Qualitative studies that focus on the use of robo advisors identified that the users require “just the right amount of transparency” meaning that there is enough comprehension of the logic of the system without needing too much detail (Zhu et al., 2023). Moreover, the implications of transparency for the users might vary based on their skills since more financially knowledgeable individuals can adequately use transparent details, while simpler explanations might be more important compared to technical explanations for those with less expertise (Zhu et al., 2023; Zhu et al., 2024).

### 2.3 Self-determination theory framework

Self-Determination Theory (SDT) is one of the significant theoretical constructs available for interpreting human behavior and motivation that has been conceptualized by researchers Deci and Ryan (Deci and Ryan, 2000). The central idea of this theory is that human beings have three basic needs: autonomy, competence, and relatedness. Fulfillment of these three needs is directly associated with an individual’s intrinsic motivation, wellbeing, and behavior.

The need for autonomy: This refers to the need for self-determination and freedom of will on the part of the individual. Autonomy is attained when people experience that they are the initiators and controllers rather than being controlled by others or external factor (Deci and Ryan, 2000). Perceived autonomy in AI-assisted decision-making scenarios emerges as an important variable: it captures the subjective experience of control by the user during the decision-making process in interacting with the algorithmic system (Puntoni et al., 2021). Research has revealed that when designing AI systems to increase the level of user freedom in decision-making and their subjective feelings of control, they will be receptive to decisions made by the algorithmic system (Fan and Liu, 2022). However, where the user perceives that their decisions are being ‘taken over by the algorithm,’ they may experience reactance (André et al., 2018).

The need for competence speaks about the need for the individual to be able to influence the environment effectively and reach desired outcomes. Talking about investment decisions, the need for competence can be identified through the investor being confident about his/her own judgment and decision-making capacities (Deci and Ryan, 2000). The social learning processes are also successful because these processes allow the investor to overcome his/her deficiency in information and skills—the investor can take cues from others, allowing him/her to make better decisions, thus boosting his/her feelings of competence (Baek and Kim, 2023).

The need for relatedness refers to the individual’s need for social connection, belonging, and being cared for. In the investment context, the need for relatedness is met through social utility mechanisms: holding the same assets as friends creates common topics of conversation, strengthens group identity, and satisfies the need for belonging (Deci and Ryan, 2000).

Applying Self-Determination Theory to the context of this study, we can derive the theoretical logic of how algorithmic transparency moderates social influence. When algorithmic recommendations have high transparency, users can understand the basis and logic of the recommendations, which enhances their sense of competence (“I understand why this investment is appropriate”) and autonomy (“I made my own decision based on sufficient information”). Increased feelings of competence and autonomy mean that users become less dependent on external information sources (including the investment behavior of friends)—they trust their own judgment more than blindly following others (Starke et al., 2022). Therefore, under conditions of high transparency, the social learning effect may be weakened.

However, the impact of transparency on the social utility mechanism may be limited. Social utility stems from the need for relatedness, focusing on the social value of maintaining connections with others, rather than information-level considerations. Even if users fully understand the logic of algorithmic recommendations and are confident in their decision-making abilities, they may still choose the same investments as their friends because they want to “go along” with them—because the social utility this brings is independent of the information value (Yang and Lee, 2024). In other words, increased autonomy and feelings of competence do not weaken the need for relatedness.

### 2.4 Hypothesis development

**Figure 1:**
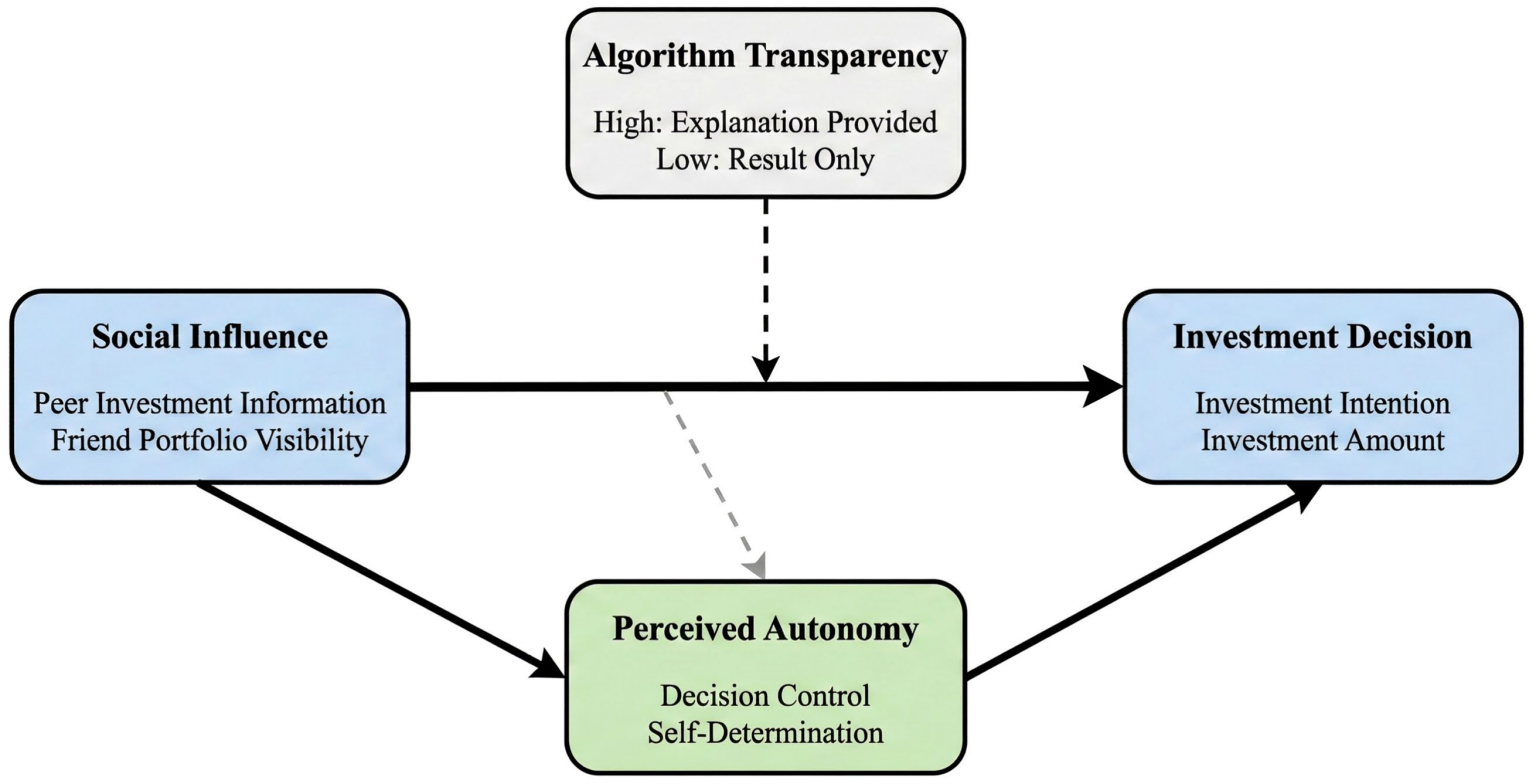
Conceptual framework of the research.

Based on the above theoretical analysis, this study constructs a theoretical model of how algorithmic transparency moderates social influence. Figure 1 presents the core conceptual framework of this study, clearly showing the logical relationship between algorithmic transparency, social influence, perceived autonomy, and investment decisions.

#### 2.4.1 The moderating effect of algorithm transparency on social learning effects

The core of the social learning mechanism lies in the alternative acquisition of information. When investors face insufficient information or limited processing capacity, the investment choices of others become an important source of signals for inferring asset quality (Puntoni et al., 2021). However, this degree of reliance on social information is not fixed, but is influenced by the richness of available information in the decision-making environment. Under low transparency conditions, algorithmic recommendations only present results without providing explanations. Investors lack the information basis for independent judgment, and thus the investment choices of friends become a key clue to fill the information gap. Conversely, when algorithmic recommendations have high transparency, the system provides detailed explanations of the basis for the recommendations—risk assessment, return prediction, matching logic, etc. This information functionally replaces the reference value that social learning can provide (Li et al., 2022; Zhang et al., 2025).

From the perspective of self-determination theory, high transparency enhances users’ sense of competence: users not only know “what is recommended,” but also understand “why it is recommended.” This understanding provides them with the cognitive resources to independently evaluate investment value (Baek and Kim, 2023). The enhancement of competence reduces investors’ reliance on external information sources—when individuals believe they are capable of making wise decisions, the motivation to follow others’ choices naturally weakens (Fan and Liu, 2022). In addition, high transparency also strengthens users’ sense of autonomy: decisions made based on sufficient information are experienced as “their own choices” rather than “guided choices,” and this sense of autonomy further weakens conformity (André et al., 2018). Therefore, this study expects that algorithm transparency will negatively moderate the social learning effect. Accordingly, the following hypothesis is proposed:

*Hypothesis 2*: Algorithm transparency negatively moderates the social learning effect. Specifically, compared to low transparency conditions, the positive impact of social information on investment intention is weaker under high transparency conditions.

#### 2.4.2 The moderating effect of algorithm transparency on social utility effects

Unlike the social learning mechanism, the driving force of the social utility mechanism is not information acquisition, but the maintenance of social connections (Bursztyn et al., 2014). To establish common themes, build group identity, and fulfill the need for feelings of belonging, investors select the same targets for investment as their friends; such social benefits are distinct from any informational utility of the investment itself. When viewed through the lenses of Self-Determination Theory, “Social utility is the equivalent of the need for relatedness rather than the needs for competence or autonomy” (Deci and Ryan, 2000). The point of theoretical divergence here is that whereas “algorithmic utility is fundamentally a cognitive process—it alleviates ignorance, improves understanding, and enables decision-making a utility of a social nature is “fundamentally an emotional/social experience—it fulfills a psychological need for connection” with others. Even when investors have a clear understanding of the logic of the algorithms’ suggested choices and are confident in their analytical skills, they may nonetheless select the same investments in an effort to fulfill a “social motive of wanting to be with friends” (Bursztyn et al., 2014). This implies that greater feelings of competency and autonomy associated with a greater sense of transparency do not reduce an individual’s need for connection with others.

This argument is indirectly supported by empirical studies. Empirical studies analyzing retirement saving decisions have found that having close social connections tends to have a greater effect on individual saving decisions in a group, and this happens mostly through interaction paths rather than information transfer paths (Duflo and Saez, 2003). This implies that if the major force of the sharing process is striving for relatedness, the factors in the information environment may not have a great effect on changing behavioral patterns efficiently. On this basis, one hypothesis may be stated:

*Hypothesis 3*: Algorithmic transparency has a weak (or insignificant) moderating effect on the social utility effect.

Given the experimental design constraints, Hypothesis 3 represents an exploratory prediction rather than a definitively testable hypothesis. The current study employs heterogeneity analysis based on self-reported decision motivation tendencies to provide preliminary evidence, acknowledging that conclusive causal testing would require direct experimental manipulation of social learning versus social utility mechanisms in future research.

#### 2.4.3 The mediating role of perceived autonomy

Based on the analysis above, the psychological mechanism underlying the transparency moderating effect can be explained as follows: transparency alters the pattern of response of investors toward social information based on perceived autonomy. Perceived autonomy can be defined as the feelings of control and autonomy that a person perceives when making a decision (Fan and Liu, 2022). With high transparency in algorithmic recommendation, users can analyze the logic of recommendation, scrutinize the grounding for the recommendation, and also generate autonomous decisions. This increases the autonomy that users perceive concerning the decision—decisions perceived as “a choice I made based on understanding” rather than “a choice the system made for me”.

In what way does perceived autonomy contribute to a weakening of social influence? According to studies in psychological research, when individuals perceive that they have enough autonomy in a certain decision, they are more likely to follow their own analysis and judgment instead of being influenced by others in making a decision. In the context of investment decisions, a high perceived autonomy condition indicates that individuals attribute their decisions to their own reason rather than to algorithmic influence or friends. In a low transparency condition, however, individuals lack understanding about the logic of the recommendation system. The decision-making process in this case depends more on external influence—at this point in time, the investment decisions of friends can be taken as understandable and trustworthy signals in a natural way.

Based on the above analysis, this study proposes perceived autonomy as a mediating mechanism for the moderating effect of transparency. Specifically, high transparency enhances perceived autonomy, and the increase in perceived autonomy reduces investors’ reliance on social information. This mediating path constitutes the psychological channel through which transparency “works.” Accordingly, the following hypothesis is proposed:

*Hypothesis 4*: Perceived autonomy mediates the moderating effect of algorithmic transparency on social influence. Specifically, high transparency reduces investors' reliance on social information by enhancing perceived autonomy.

**Figure 2:**
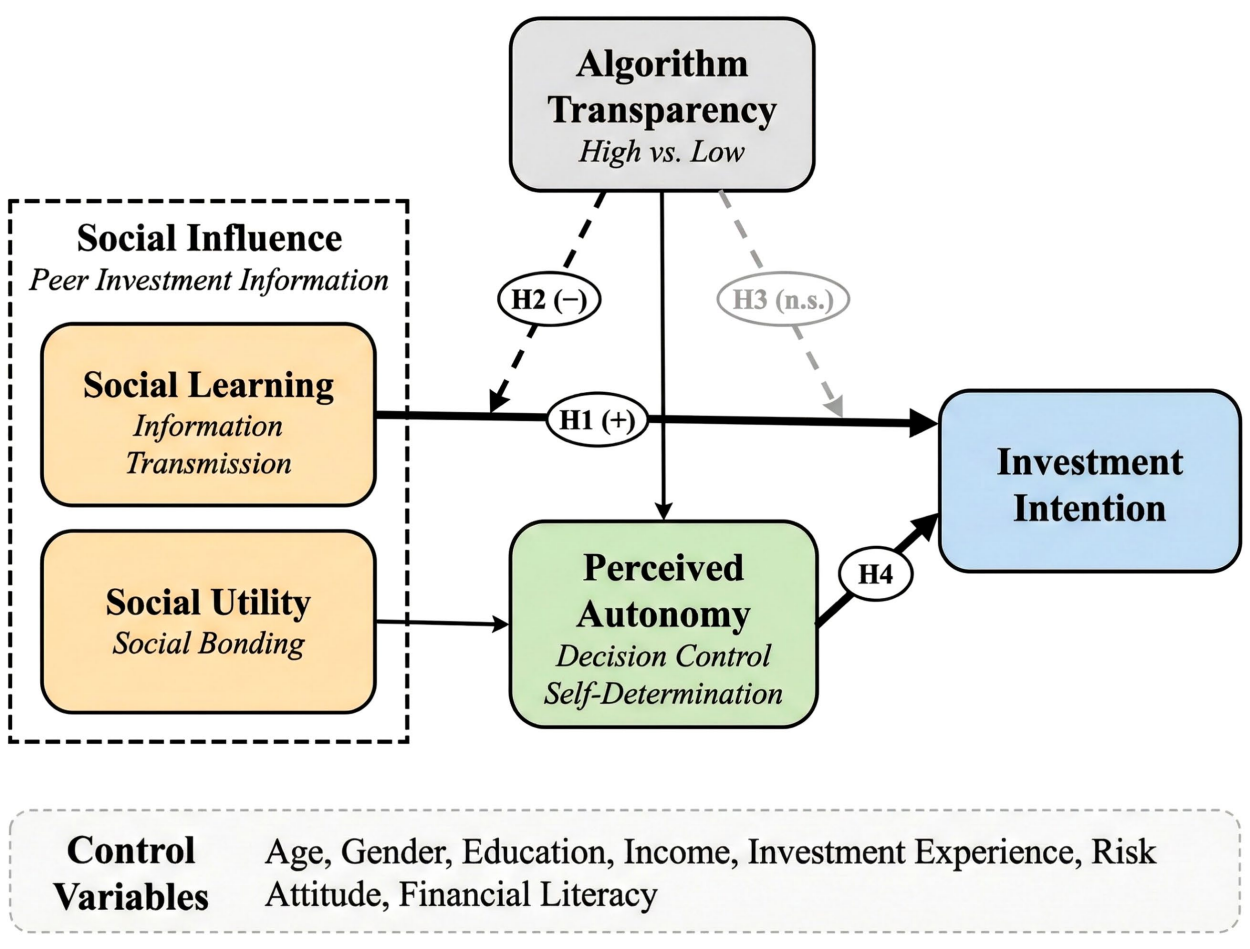
Research model and hypotheses. Solid lines indicate direct effects; dashed lines indicate moderating effects; H1, H2, H3, and H4 represent hypothesized relationships; (+), positive effect; (−), negative effect; n.s., non-significant.

As shown in Figure 2, the complete research model and hypothesized paths of this study are presented. Social influence (H1) positively affects investment intention; algorithmic transparency negatively moderates this effect (H2), and this moderation is mainly reflected in the social learning mechanism, with a weaker expected moderating effect on the social utility mechanism (H3); perceived autonomy plays a mediating role in the transparency moderating effect (H4). Solid lines represent direct effects, and dashed lines represent moderating effects. Control variables include age, gender, education level, income, investment experience, risk attitude, and financial literacy.

## 3 Research methods

### 3.1 Overview of research design

**Figure 3:**
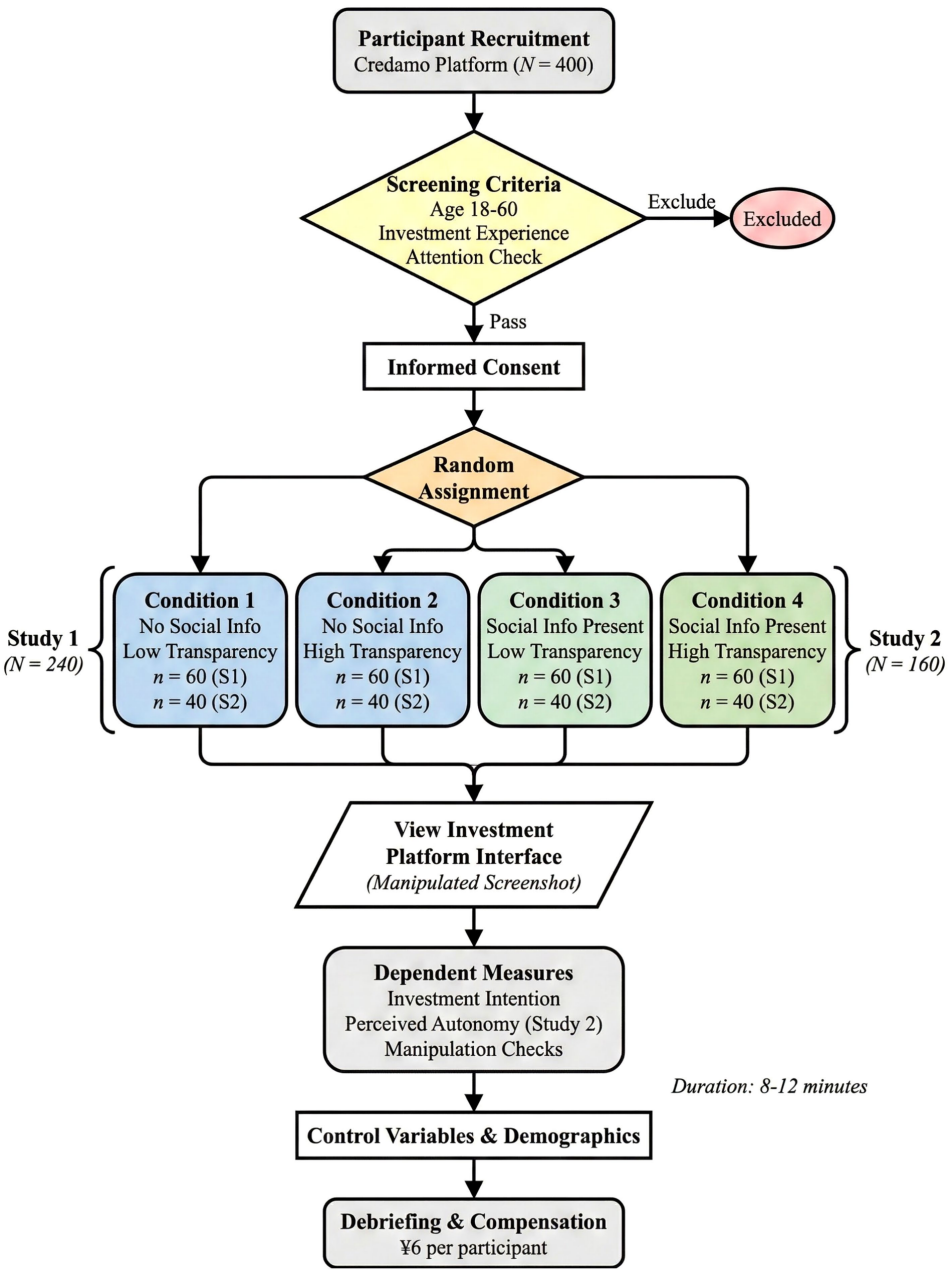
Experimental procedure flowchart. S1, Study 1; S2, Study 2.

To systematically test the above hypotheses, this study employed two progressive online experiments. Study 1 used a 2 (Social Information: Present/Absent) × 2 (Algorithm Transparency: High/Low) between-subjects experimental design to test the main effect of social influence (H1) and the moderating effect of algorithm transparency (H2, H3). Study 2 was an extension of Study 1 in the sense that the study added the assessment of the mediating variables to examine the mediating effects of autonomy perception (H4). For both studies, the same experimental paradigm was employed. As can be seen in Figure 3, the experiment involved screening for participants before random allocation to the experimental manipulation phases.

### 3.2 Participants and sample

The study was approved by the Ethics Committee of Capital University of Economics and Business (Approval No. CUEB-2024-212). All participants provided written informed consent prior to participation. Participants were recruited through the Credamo platform (see questionnaire). Credamo is a leading online research platform in China with over 3 million registered users and is widely used in consumer behavior and financial decision-making research. Sample selection criteria included: age 18–60 years; experience in fund or stock investment (investment activity within the past year); and passing attention check questions (trap questions embedded in the questionnaire). Study 1 recruited 240 participants, randomly assigned to four experimental conditions (60 participants per group). Study 2 recruited 160 participants, randomly assigned to four experimental conditions (40 participants per group). The total sample size was 400. The sample size was determined based on the effect size of previous similar studies (*d* ≈ 0.4) and statistical power analysis (1−*β* = 0.80, *α* = 0.05), with 60 participants per group being sufficient to detect medium effect sizes for main effects and interaction effects. Table 1 presents the experimental design and sample allocation for the two studies.

**Table 1:** Experimental design and sample allocation.

Condition	Social information	Algorithm transparency	Study 1 sample	Study 2 sample
Condition 1	Absent	Low	60	40
Condition 2	Absent	High	60	40
Condition 3	Present	Low	60	40
Condition 4	Present	High	60	40
Total	–	–	240	160

### 3.3 Experimental procedure

After providing informed consent, participants were informed that they would be participating in a survey about their experience using an investment platform. Participants were subsequently allotted to four different conditions of the experiment through random assignment. Each was shown a screenshot of a hypothetical investment website interface. The interface contained recommendations for a balanced fund. It also contained data like the name of the fund, the return rate for the most recent period, and the risk rating.

Social Information Manipulation: In the “with social information” condition, the interface showed “3 of your friends have invested in this fund,” accompanied by the avatars and investment amounts of the friends. In the “without social information” condition, the interface did not display this information. Algorithm Transparency Manipulation: In the “high transparency” condition, the interface showed the recommendation reason in a designated place at the bottom of the interface: “Based on your risk tolerance (medium) and investment horizon (3–5 years), in combination with the fund’s historical return rate (12.3% per annum over the past 3 years), volatility (standard deviation 15.2%), Sharpe Ratio (0.81), the system recommends this fund after comprehensive evaluation.” In the “low transparency” condition, there was no explanation underneath the “smart recommendation” text.

After reading the experimental materials, participants completed measures of the dependent variable (investment intention), the mediating variable (perceived autonomy, only in Study 2), manipulation check questions, and control variables (demographic variables, risk attitude, financial literacy). The experiment lasted approximately 8–12 min, and participants received a 6 RMB reward.

### 3.4 Variable measurement

Dependent Variable: Investment Intention. Measured using a three-item scale, adapted from established scales in relevant research. The items included: “I am willing to invest in this fund,” “I intend to learn more about this fund,” and “If I had spare funds, I would consider buying this fund.” A 7-point Likert scale was used (1 = strongly disagree, 7 = strongly agree). The Cronbach’s *α* coefficient of the scale was 0.89 (Study 1) and 0.91 (Study 2), indicating good internal consistency.

Mediating Variable: Perceived Autonomy (only in Study 2). Measured using a four-item scale, adapted from relevant research. The items included: “When making investment decisions, I feel I have sufficient freedom of choice,” “I feel that this investment decision was made by myself,” “I feel that I can control the investment decision-making process,” and “I feel that I am not forced to accept the system’s recommendations.” A 7-point Likert scale was used. The Cronbach’s α coefficient of the scale was 0.87.

Control variables included: age (continuous variable), gender (0 = female, 1 = male), education level (1 = high school or below, 2 = junior college, 3 = bachelor’s degree, 4 = master’s degree or above), monthly income (1 = below 5,000 RMB, 2 = 5,000–10,000 RMB, 3 = 10,000–20,000 RMB, 4 = above 20,000 RMB), investment experience (1 = less than 1 year, 2 = 1–3 years, 3 = 3–5 years, 4 = more than 5 years), risk attitude (single item, “What do you think your investment style is?” 1 = very conservative, 7 = very aggressive), and financial literacy (three objective questions measuring understanding of compound interest, inflation, and risk diversification, score 0–3).

Decision motivation tendency. To distinguish the roles of different types of social influence mechanisms (for exploratory analysis), this study used two single-item measures of participants’ decision motivation: information seeking tendency (“When making investment decisions, I mainly focus on obtaining sufficient information and analysis”) and social needs tendency (“When making investment decisions, I will consider my friends’ investment choices to maintain social relationships”). A 7-point Likert scale was used (1 = strongly disagree, 7 = strongly agree).

### 3.5 Analysis strategy

The following methods were used for data analysis: manipulation checks used chi-square tests to verify the effectiveness of the experimental manipulation; main effects and interaction effects were analyzed using a 2 × 2 two-way ANOVA to test the main effects and interaction effects of social information and algorithm transparency; simple effects analysis was conducted when the interaction effect was significant, further analyzing the simple effects of social information under different transparency conditions; mediation analysis used Hayes’ PROCESS macro program (Model 8) to test the mediating role of perceived autonomy. Bootstrap sampling was set to 5,000 times, and the confidence interval was 95%. The criterion for judging the mediating effect was that the Bootstrap confidence interval did not include zero. All analyses were performed using SPSS 26.0 and R 4.2.0 software. The significance level was set at *α* = 0.05.

## 4 Research results

### 4.1 Sample characteristics and manipulation check

Sample characteristics. Of the 240 participants in Study 1, 52.1% were male, with an average age of 32.4 years (SD = 8.7), 71.3% had a bachelor’s degree or higher, and the average investment experience was 2.4 years. The characteristics of the 160 participants in Study 2 were similar to those in Study 1, with 49.4% male, an average age of 31.8 years (SD = 9.2), and 68.8% having a bachelor’s degree or higher.

Table 2 presents the demographic characteristics and individual difference variables across the four experimental conditions. One-way ANOVA tests for continuous variables and chi-square tests for categorical variables confirmed that random assignment achieved satisfactory group equivalence, with no statistically significant between-condition differences detected for any measured variable (all *p* > 0.05; Table 2). These results confirm that the observed experimental effects cannot be attributed to systematic pre-existing group differences.

**Table 2:** Demographic characteristics and individual differences across experimental conditions (Study 1).

Variable	SI−/AT−	SI−/AT+	SI+/AT−	SI+/AT+	*F*/*χ*^2^	*p*
*n*	60	60	60	60	–	–
Age (*M* ± SD)	31.8 ± 8.5	32.9 ± 9.0	33.1 ± 8.9	31.8 ± 8.4	0.38	0.767
Gender (% female)	55.0%	50.0%	45.0%	40.0%	3.01	0.390
Education					6.58	0.361
High school or below	20.0%	16.7%	15.0%	10.0%		
Bachelor’s degree	63.3%	65.0%	55.0%	60.0%		
Master’s or above	16.7%	18.3%	30.0%	30.0%		
Monthly income					5.24	0.514
< ¥5,000	33.3%	38.3%	35.0%	30.0%		
¥5,000–10,000	35.0%	31.7%	33.3%	36.7%		
¥10,000–20,000	21.7%	20.0%	23.3%	21.7%		
> ¥20,000	10.0%	10.0%	8.3%	11.7%		
Invest. exp. (*M* ± SD)	2.87 ± 0.87	3.18 ± 0.83	2.78 ± 0.95	2.98 ± 0.91	2.27	0.081
Risk attitude (*M* ± SD)	3.94 ± 1.13	4.17 ± 1.27	3.85 ± 1.13	4.01 ± 1.15	0.79	0.501
Fin. literacy (*M* ± SD)	2.05 ± 0.75	1.90 ± 0.77	1.93 ± 0.78	2.10 ± 0.82	0.88	0.450

SI, Social Information (−, absent, +, present); AT, Algorithmic Transparency (− = low, + = high). Continuous variables tested with one-way ANOVA (*F*); categorical variables tested with *χ*^2^ test. No significant between-condition differences were detected (all *p* > 0.05, all *η*^2^ < 0.03, all Cramér’s *V* < 0.12).

Manipulation check. In the “with social information” condition, 89.2% of participants correctly identified that friend investment information was displayed on the interface; in the “without social information” condition, the correct identification rate was 91.7% (*χ*^2^ = 156.83, *p* < 0.001). The algorithm transparency manipulation was successful: in the “high transparency” condition, 84.2% of participants reported seeing the recommendation reasons; in the “low transparency” condition, the correct identification rate was 87.5% (*χ*^2^ = 98.45, *p* < 0.001). Although the identification rate for the transparency manipulation was slightly lower than that for the social information manipulation, both manipulations met acceptable validity standards. Table 3 presents the descriptive statistics for the main variables.

**Table 3:** Descriptive statistics (Study 1, *N* = 240).

Variable	Mean	SD	Min	Max
Investment intention	4.68	1.24	1.33	7.00
Risk attitude	3.82	1.35	1	7
Financial literacy	1.87	0.92	0	3
Age	32.4	8.7	18	58
Investment experience (years)	2.4	1.6	0.5	12

Although the current manipulation check employed categorical identification items rather than a continuous perceived transparency scale, the high correct identification rates in both conditions, combined with the substantial chi-square statistics, provide converging evidence that the binary transparency manipulation achieved its intended effect of creating distinctly different information environments for participants across experimental conditions.

### 4.2 Testing the main effect of social influence (H1)

The ANOVA results of Study 1 showed a significant main effect of social information (*F*(1,236) = 24.87, *p* < 0.001, *η*^2^ = 0.095). Investment intention under the social information condition (*M* = 5.12, SD = 1.18) was significantly higher than under the no social information condition (*M* = 4.24, SD = 1.21), with a mean difference of 0.88 units (regression coefficient *B* = 0.85, SE = 0.16, *p* < 0.001, see Table 4). Hypothesis 1 is supported. Table 4 presents the detailed regression results of the main effect of social influence.

**Table 4:** Main effect regression results (Study 1).

Predictor	*B*	SE	*t*	*p*	95% CI
Constant	3.42	0.45	7.60	<0.001	[2.54, 4.30]
Social information (Present = 1)	0.85	0.16	5.31	<0.001	[0.54, 1.16]
Age	−0.01	0.01	−1.00	0.318	[−0.03, 0.01]
Gender (Male = 1)	0.18	0.15	1.20	0.231	[−0.12, 0.48]
Risk attitude	0.22	0.06	3.67	<0.001	[0.10, 0.34]
Financial literacy	0.09	0.08	1.13	0.260	[−0.07, 0.25]
Investment experience	0.06	0.05	1.20	0.231	[−0.04, 0.16]
*R* ^2^	0.142				
*F*	6.52^***^				

This table presents the results of the main effects model without interaction terms. *p < 0.05; **p < 0.01; ***p < 0.001.

### 4.3 Test of the moderating effect of algorithm transparency (H2, H3)

**Figure 4:**
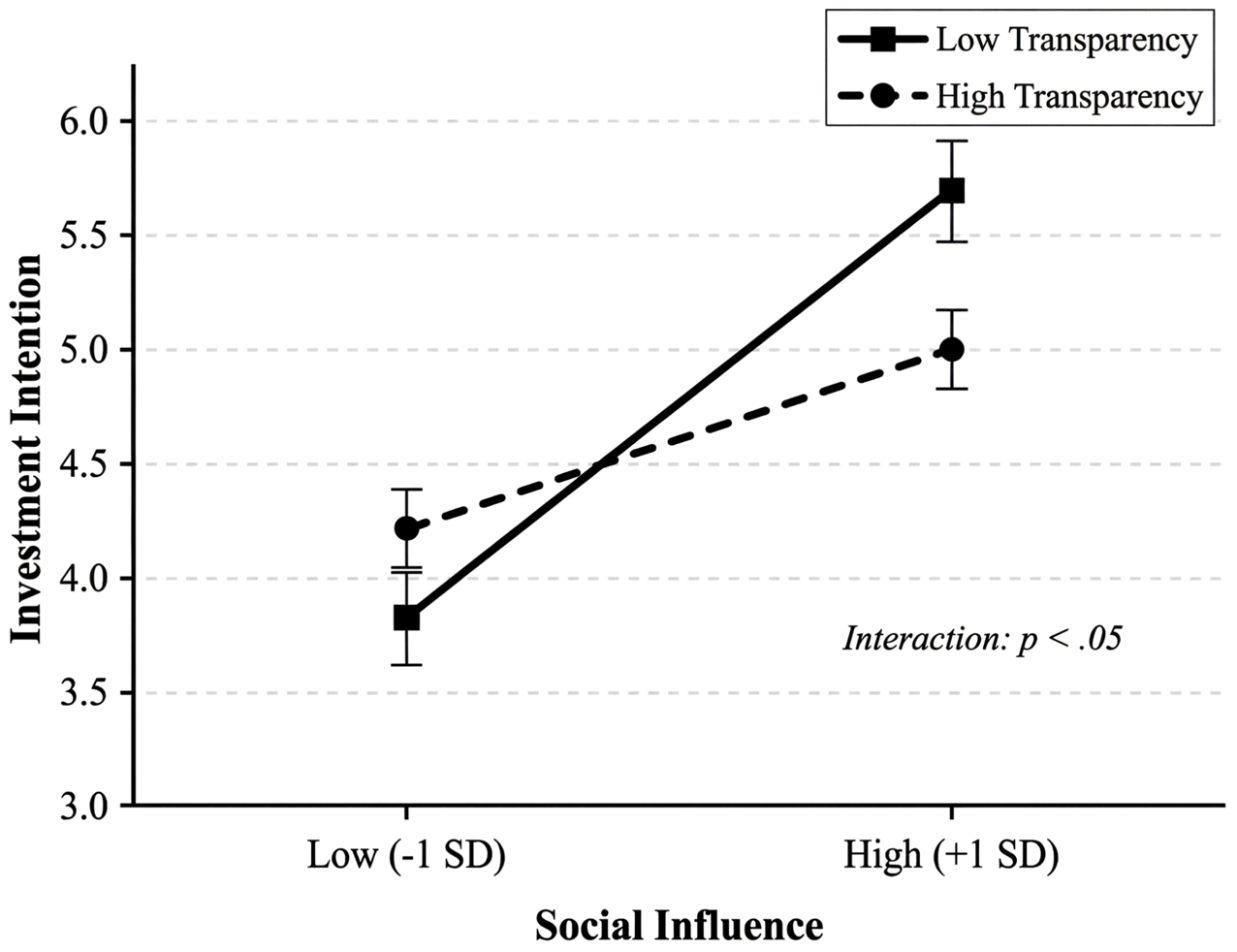
Interaction effect of social influence and algorithm transparency on investment intention. Error bars represent 95% confidence intervals.

Two-way ANOVA showed a significant interaction effect between social information and algorithm transparency (*F*(1,236) = 4.89, *p* = 0.028, *η*^2^ = 0.020). This indicates that algorithm transparency does moderate the effect of social information on investment intention. As shown in Figure 4, under low transparency conditions, the investment intention of the group with social information (*M* = 5.09, SD = 1.12) was significantly higher than that of the group without social information (*M* = 3.85, SD = 1.15), with a difference of 1.24 units (simple effect: *F*(1,236) = 18.76, *p* < 0.001). Under high transparency conditions, the investment intention of the group with social information (*M* = 5.02, SD = 1.24) was also higher than that of the group without social information (*M* = 4.21, SD = 1.18), but the difference was only 0.81 units (simple effect: *F*(1,236) = 6.12, *p* = 0.014). Calculating the moderating effect size, the effect of social information under high transparency conditions decreased by approximately 34.7% compared to low transparency conditions (from 1.24 to 0.81). This result supports Hypothesis 2: Algorithm transparency negatively moderates the social learning effect, and the facilitating effect of social information on investment intention is significantly weakened under high transparency conditions. Table 5 presents the regression analysis results including the interaction effect.

**Table 5:** Moderation effect regression results.

Predictor	*B*	SE	*t*	*p*
Constant	3.28	0.46	7.13	<0.001
Social information (SI)	1.78	0.22	8.09	<0.001
Algorithm transparency (AT)	0.38	0.22	1.73	0.085
SI × AT	−0.89	0.31	−2.87	0.004
Control variables	Included			
*R* ^2^	0.186			
Δ*R*^2^ (Interaction)	0.028^**^			

This table presents the moderation model with interaction term. The coefficient for Social Information (SI) represents the simple effect under low transparency condition. *p < 0.05; **p < 0.01; ***p < 0.001.

While the interaction effect achieves statistical significance (*p* = 0.028), the effect size is relatively small (*η*^2^ = 0.020) according to Cohen (2013) benchmarks (small = 0.01, medium = 0.06, large = 0.14), warranting careful interpretation regarding practical significance. Several design factors may contribute: the hypothetical scenario likely reduced motivational salience compared to real financial decisions; the binary transparency manipulation may have compressed natural variance in perceived transparency; and static screenshots lacked dynamic platform features that might amplify transparency effects. Nonetheless, even small effects can accumulate meaningful practical significance in high-frequency decision contexts—a consistent 34.7% reduction in social conformity bias could translate into substantial aggregate improvements in portfolio diversification and decision quality over time. Field studies employing actual transaction data and dynamic platform interfaces may reveal larger effect sizes.

To test Hypothesis 3 (that transparency has a weak moderating effect on the social utility mechanism), this study conducted an exploratory heterogeneity analysis. Based on the median of decision-making motivation tendencies, participants were divided into a “high information needs group” (*n* = 118, more focused on information acquisition) and a “high social needs group” (*n* = 122, more focused on social connection). The results of the group regression showed that in the high information needs group, the moderating effect of transparency was significant (interaction term *β* = −0.92, *p* = 0.008); in the high social needs group, the moderating effect was weaker and not significant (interaction term *β* = −0.38, *p* = 0.156). It should be noted that this heterogeneity analysis is an exploratory test and has the following limitations: (1) Decision-making motivation is a characteristic of the participants rather than an experimental manipulation, so causal inference cannot be established; (2) The difference in moderating effects between the two groups did not reach statistical significance (Δ*β* = −0.54, *p* = 0.089). Therefore, while the pattern of results aligns with theoretical predictions, Hypothesis 3 receives only tentative empirical support. The exploratory heterogeneity analysis based on median splits reveals directionally consistent findings (stronger transparency moderation in the high information needs group: *β* = −0.92, *p* = 0.008; weaker moderation in the high social needs group: *β* = −0.38, *p* = 0.156), though the between-group difference does not achieve statistical significance (Δ*β* = −0.54, *p* = 0.089). These results provide preliminary evidence consistent with theoretical predictions, yet conclusive validation requires future research employing direct experimental manipulation of the two distinct social influence mechanisms—for instance, by framing social information differentially as asset quality signals (informational framing) versus relational cues (social framing).

### 4.4 Mediation effect test of perceived autonomy (H4)

**Figure 5:**
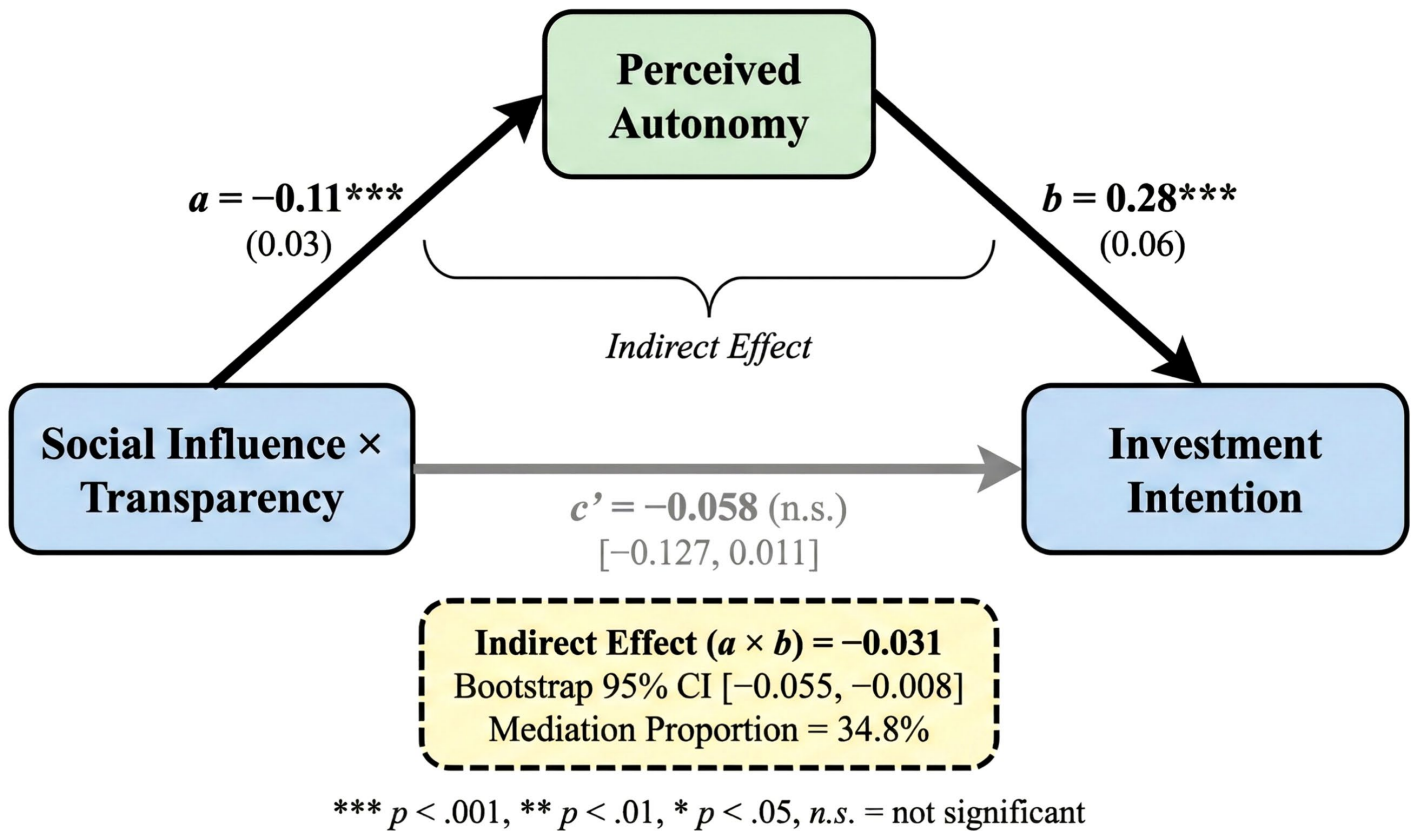
Mediation effect of perceived autonomy (bootstrapping results). Unstandardized coefficients are reported; values in parentheses are standard errors; ***p < 0.001; n.s., not significant; bootstrap sample = 5,000.

The data from Study 2 were used to test the mediating effect of perceived autonomy. PROCESS Model 8 was used for the moderated mediation analysis, with a Bootstrap sample size of 5,000. As shown in Figure 5, the analysis results indicate that: the interaction term between social information and algorithmic transparency has a significant negative impact on perceived autonomy (path a: *B* = −0.11, SE = 0.03, *p* < 0.001); perceived autonomy has a significant positive impact on investment intention (path b: *B* = 0.28, SE = 0.06, *p* < 0.001); after controlling for the mediating variable, the direct effect of the interaction term on investment intention becomes insignificant (path c′: *B* = -0.058, SE = 0.035, 95% CI [−0.127, 0.011]). The bootstrap test of the indirect effect (a × b) showed that the indirect effect value was −0.031, with a 95% confidence interval of [−0.055, −0.008], which does not include zero, indicating that the mediating effect is significant. The mediation proportion was 34.8%, meaning that approximately one-third of the moderating effect of algorithm transparency is transmitted through perceived autonomy. Table 6 presents the detailed results of the mediation analysis.

**Table 6:** Mediation analysis results (Study 2, *N* = 160).

Path	Effect	SE	95% CI
a path (SI × AT → PA)	−0.110^***^	0.030	[−0.170, −0.050]
b path (PA → II)	0.280^***^	0.060	[0.160, 0.400]
Direct effect (c′)	−0.058	0.035	[−0.127, 0.011]
Indirect effect (a × b)	−0.031^*^	0.012	[−0.055, −0.008]
Mediation proportion	34.8%	–	–

SI, Social Information; AT, Algorithm Transparency; PA, Perceived Autonomy; II, Investment Intention.

^*^p < 0.05. ^**^p < 0.01. ^***^p < 0.001.

This result partially supports Hypothesis 4: perceived autonomy plays a partial mediating role in the moderating effect of algorithmic transparency. High transparency reduces users’ reliance on social information by enhancing their perceived autonomy, thereby weakening the social influence effect. The fact that the direct effect becomes insignificant indicates that most of the transparency moderating effect can be explained by perceived autonomy.

### 4.5 Robustness tests

To ensure the robustness of the results, this study carried out a series of sensitivity analyses. When financial literacy, risk preference, and investment experiences were added as covariates in the analysis, the original results were confirmed again: the interaction effect remained statistically significant (*F*(1, 232) = 4.52, *p* = 0.034), and the strength of the effect slightly decreased (*η*^2^ = 0.018). As the normality assumption may not hold for the dependent variable, an alternative test employing the Kruskal-Wallis test statistic was carried out for re-analysis. Comparing the four groups, there were statistically significant differences (H(3) = 28.67, *p* < 0.001), and again the pairwise comparison results were consistent with the original test statistic. When investors whose task completion times were shorter than 3 min and longer than 20 min were excluded (5.2% exclusions), the re-analysis confirmed that there were no major differences in the results. To test whether other mediating processes could exist, “perceived trust” and “perceived usefulness” were examined alternatively as the mediating variables.

As hypothesized, the mediating effect of “perceived autonomy” remains statistically significant and not influenced by either “perceived trust” or “perceived usefulness.” To investigate boundary conditions, exploratory subgroup analyses were conducted across theoretically relevant participant characteristics. Financial literacy was selected as the primary stratification variable given its conceptual relevance to the information-processing mechanism underlying the transparency moderating effect. Among participants with low-to-moderate financial literacy (score ≤ 2, *n* = 172), the interaction effect remained statistically significant and consistent with the full-sample pattern. Supplementary analyses stratifying by investment experience and risk attitude yielded no significant interaction effects, suggesting that financial literacy represents the most meaningful boundary condition in the present dataset.

Furthermore, subgroup analysis reveals an important boundary condition regarding investor sophistication. When re-analyzing the high-financial-literacy subgroup (financial literacy score = 3, *n* = 68), the negative moderating effect of transparency became statistically non-significant (interaction term *β* = −0.52, *p* = 0.118), indicating that the core effects identified in this study are primarily applicable to nonprofessional retail investors rather than financially sophisticated participants. High-literacy investors likely possess sufficient domain expertise and analytical capabilities to independently evaluate investment quality without substantial reliance on either social cues or algorithmic explanations, thereby attenuating both the baseline social influence effect and the transparency moderating effect. For this investor segment, variations in information environment characteristics exert diminished influence on decision-making patterns. These boundary conditions suggest that the findings hold particular relevance for the majority nonprofessional investor population characterized by limited financial knowledge. Such boundary conditions imply that the results of this study may better apply to non-professional investors with low to medium levels of financial literacy. From the above sensitivity test results, the central result that algorithmic transparency negatively moderates the social learning effect and that “perceived autonomy” serves as the mediating effect remains robust for the original sample, but with certain boundary conditions with respect to demographic characteristics.

## 5 Discussion

### 5.1 Main findings

This study specifically analyzed the role of transparency of algorithms as a moderator of social influence in investment choices in two online studies. Social influence proves to have a strong positive effect on investment intention. When investors are presented with information through the interface of the platform about the previous investment of their social connections in a certain fund, there is a significant increase in investment intention. This result is in line with many other studies in the past, which verifies once again the universality of the role of social influence in investment choices, in this case, in the context of an intelligent investment advice online platform (Chen, 2024; D’Acunto et al., 2019).

The transparency of algorithmic processes greatly reduces the strength of the social learning effect. In a low transparency setting, the social information influence on investment intention is greater; in a high transparency setting, this influence is significantly dampened, with a decrease of about 34.7% (from 1.24 to 0.81). In this result, a critical practical implication is discovered—that is, transparency can weaken blindly following others in investment decision-making (Grimmelikhuijsen, 2023).

The moderation of the social influence mechanism by transparency is not consistent. Conforming to theoretical expectations, heterogeneity analysis finds that the moderating effect of transparency is stronger in the case of investors driven by information acquisition motives and is weaker in the case of investors driven by a social connection motive. These findings validate theoretical predictions made in this study and conclude that transparency plays a moderating role in the social learning mechanism regulated through perceived competence and autonomy but not in social utility mechanisms based on relatedness desires (Mahmud et al., 2022).

Perceived autonomy has a partial mediating effect. As shown by results from mediation analysis, algorithm transparency decreases users’ reliance on social information through increasing their perceived autonomy. The value of the mediation proportion is 34.8%, suggesting perceived autonomy plays an important role as the inner mechanism for moderation effect brought about by transparency (Fan and Liu, 2022).

### 5.2 Theoretical contributions

This study integrates two relatively independent research areas: algorithm transparency and social influence. Previous studies have mostly focused on the direct effects of transparency on user trust and adoption behavior, or the independent role of social influence on financial decision-making, but have rarely examined their interaction. This study finds that transparency not only affects users’ responses to algorithm recommendations themselves but also moderates their processing of other information sources (social information). This finding expands our understanding of the interaction of multiple information sources in a digital decision-making environment (Puntoni et al., 2021).

Research on the heterogeneity of peer effects is continued. By introducing two distinct social learning processes—information-driven social learning processes and socially driven social utility processes—research outcomes show the selective character of the transparency moderation effect. It is established that the transparency moderation effect is more of a moderator of the information-driven social learning processes rather than the socially driven social utility processes. The aforementioned has opened up new avenues for the explanation of the heterogeneity of peer effects. Where the decision-making environment is sufficiently furnished with alternative information sources (such as in the presence of transparent algorithmic recommendations), the social learning value drops, consequently reducing the peer effects (Glikson and Woolley, 2020).

The paper further expands the use of self-determination theory with regard to AI-assisted decision-making processes. Through the correlation of autonomy, competence, and relatedness needs with algorithmic transparency, social learning, and social utility mechanisms, this paper constructs a conceptual framework that identifies the transparency moderation effect. Empirical confirmation of the mediating effect empowered by perceived autonomy certainly affirms the use of self-determination theory with respect to human-computer interaction processes.

### 5.3 Practical implications

Findings of this study have significant implications for platform design and regulation policies for fintech platforms. On matters concerning platform design, transparency design should be able to consider the motivations for decision-making for users. In regard to independent decision-makers with a basic motive of information gain, a high level of transparency could encourage independence and self-confidence in decision-making, and avert blind imitation of investment decisions made by others. The mediation analysis results further indicate that the effectiveness of transparency in reducing social conformity operates primarily through the psychological channel of perceived autonomy rather than through informational content alone, which suggests that platform transparency features should be designed as autonomy-enhancing tools that actively foster the subjective experience of decision control and self-determination, for instance by framing algorithmic explanations in ways that emphasize the user’s role as the ultimate decision-maker rather than presenting recommendations as prescriptive directives.

However, for users who value social connections, the impact of transparency is limited—they may still tend to choose the same investment products as their friends. Therefore, platforms can consider providing differentiated transparency presentations for different types of users. In addition, the presentation and placement of algorithmic recommendation information and social information (such as friends’ holdings) may need to be designed separately to help users more clearly understand and utilize these two types of information (Li T. et al., 2023b). Given the boundary conditions identified in robustness analyses, platform designers should consider implementing differentiated transparency strategies calibrated to user sophistication levels. For nonprofessional retail investors constituting the majority user base, high transparency interfaces featuring detailed algorithmic explanations and risk disclosures can effectively enhance decision autonomy and mitigate blind social conformity. Conversely, for professional investors or users demonstrating high financial literacy, elaborate transparency features may prove less critical given their existing independent analytical capabilities and reduced susceptibility to social influence. Adaptive transparency systems that dynamically adjust explanation detail based on assessed user proficiency could optimize the balance between regulatory compliance and user experience across heterogeneous investor populations.

For regulators, this study provides empirical evidence supporting transparency requirements (Piotrowski and Orzeszko, 2023). Transparency policies help reduce investors’ blind herd behavior, which is consistent with the policy objective of investor protection. However, regulators also need to recognize the boundaries of the transparency effect: it may not change investment behavior based on social motivations, and a certain degree of peer effect may be an inherent social need of investors, rather than a bias that can be “corrected” through information disclosure. Furthermore, overly complex transparency requirements may lead to information overload, thereby reducing the effectiveness of the policy. Therefore, regulatory policies need to strike a balance between promoting transparency and maintaining information simplicity (Zhang et al., 2024).

### 5.4 Research limitations and future directions

This study has the following limitations. Methodologically, this study employed online experiments with static interface screenshots and hypothetical investment scenarios, which ensured high internal validity but limited ecological validity. First, the dependent variable measured investment intention rather than actual investment behavior. Although intention is a well-validated predictor of behavior, the intention-behavior gap is well-documented in financial decision-making contexts involving real monetary stakes and risk exposure. Second, static screenshots cannot replicate the dynamic, interactive features of real fintech platforms—such as real-time data updates and multi-step decision processes—which may amplify both social influence and transparency effects through active user engagement. Third, the absence of real financial consequences likely attenuated motivational engagement across all experimental conditions. Future research should prioritize field experiments through collaboration with operating fintech platforms, implementing randomized A/B testing of transparency features while tracking actual investment transactions. Combining objective behavioral data with post-decision surveys measuring perceived autonomy would enable methodological triangulation between behavioral outcomes and psychological mechanisms.

Concerning sample composition, participants were exclusively recruited from the Chinese Credamo platform, introducing cultural homogeneity that limits cross-cultural generalizability. Collectivist cultural values prominent in Chinese society may systematically amplify social influence effects relative to individualistic cultural contexts, as conformity to peer norms and maintenance of social harmony carry greater normative weight in collectivist cultures. This cultural orientation may strengthen both social learning mechanisms (through elevated trust in peer information) and social utility mechanisms (through heightened relatedness needs). Furthermore, Chinese investors may exhibit distinct attitudes toward algorithmic transparency shaped by differential regulatory environments and baseline disclosure expectations compared to Western markets. The observed moderating effect of algorithmic transparency on social influence may therefore manifest differently in individualistic societies where autonomy is more culturally prioritized.

Cross-cultural replication studies employing comparable experimental designs across culturally diverse contexts are essential to establish the boundary conditions of these findings. Specifically, comparative cross-cultural designs could examine whether algorithmic transparency exhibits stronger moderating effects in individualistic societies where autonomy is culturally prioritized, or alternatively, whether collectivist cultures’ stronger baseline conformity norms amplify the transparency effect by creating greater tension between autonomous decision-making and social conformity. Multi-country studies employing identical experimental protocols across culturally diverse samples would provide crucial insights into cultural boundary conditions. In terms of hypothesis testing, this study did not directly manipulate the distinction between social learning and social utility mechanisms, and the support for H3 mainly comes from exploratory heterogeneity analysis, with limited strength of evidence. Furthermore, the moderating effect of transparency was not significant in the high financial literacy group, suggesting that there may be boundaries to the applicability of the study’s conclusions. Future research should employ direct experimental manipulation to definitively test Hypothesis 3. One approach would frame social information differentially: an ‘informational framing’ condition presenting friends’ investments as asset quality signals, versus a ‘social framing’ condition emphasizing relational aspects of coordinated investment behavior. Such designs would enable causal identification of whether algorithmic transparency has differential moderating effects on these theoretically distinct pathways. Additionally, future research should employ multi-item validated scales for decision motivation tendencies to enable the continuous moderation analysis that single-item measures in the current study could not reliably support.

From a theoretical perspective, this study focused exclusively on perceived autonomy as the mediating mechanism linking algorithmic transparency to reduced social influence, consistent with prior research emphasizing autonomy as the primary pathway through which transparency operates. However, Self-Determination Theory posits that competence and relatedness constitute equally fundamental psychological needs alongside autonomy. The current study did not measure perceived competence, defined as users’ confidence in their ability to understand and act upon algorithmic recommendations. Conceptually, high algorithmic transparency should enhance perceived competence by demystifying recommendation logic, and this enhanced competence may independently reduce reliance on social cues. The absence of a competence measure represents an incomplete test of the full SDT framework. Future research should incorporate validated measures assessing all three SDT needs to comprehensively map the psychological pathways through which transparency influences social conformity. Parallel mediation models could test whether autonomy and competence operate as independent or synergistic mediators.

As for measurement, this experiment investigated levels of transparency with a manipulation, as opposed to a measurement of transparency on a certain scale. Though this was an improvement for the validity of causal inference methodologies in this research, this method does not and can never fully account for a process of different levels of transparency and their continuous effects. Future studies could construct a transparency perception scale and explore a dose–response relation between transparency of different degrees. As for mechanism exploration, this research investigated transparency through autonomy as a mediating factor. But transparency could play various roles as a psychological mechanism of cognitive load or information process or even emotional perspectives on algorithms. Future studies could continue to explore and expand on research into mediating mechanisms. Future studies could also explore long-term and learning effects; investors’ transparency-processing capabilities may increase through practice and experience, and a moderating role of transparency could change over time (Mahmud et al., 2022).

## 6 Conclusion

On the basis of self-determination theory, this study examined the role of algorithmic transparency in moderating social influence on investment decisions in two online studies. The findings showed that social influence (investment information from friends) had a significant positive impact on investment intention, and algorithmic transparency negatively moderated this process. The moderating role is essentially shown in the social learning process. The role of perceived autonomy is a part of the mediating process in the regulatory role—higher transparency reduces reliance on social information by increasing the perceived autonomy in decision-making. This study explains the psychological processes at work in the interaction between algorithmic and social variables in the digital investment space and presents an empirical basis for the design and regulatory guidelines of FinTech platforms. The current study may be extended in future studies by conducting field studies on cross-cultural settings and by doing longitudinal studies.

## Data Availability

The original contributions presented in the study are included in the article/supplementary material, further inquiries can be directed to the corresponding author.
